# Mitochondrial development and remodeling occur at pancreatic progenitor stage during induction of stem cell-derived islet organoids

**DOI:** 10.3389/fendo.2026.1795738

**Published:** 2026-04-23

**Authors:** Abdoulaye Diane, Razik Mu-u-min, Noora Ali Al-Shukri, Wared Nour-Eldine, Heba Hussain Al-Siddiqi

**Affiliations:** 1 Diabetes Research Center, Qatar Biomedical Research Institute (QBRI), Hamad Bin Khalifa University (HBKU), Qatar Foundation (QF), Doha, Qatar; 2 Neurological Disorders Research Center, Qatar Biomedical Research Institute (QBRI), Hamad Bin Khalifa University (HBKU), Doha, Qatar; 3 College of Health & Life Sciences, Hamad Bin Khalifa University (HBKU), Qatar Foundation (QF), Doha, Qatar

**Keywords:** diabetes, differentiation, GSIS, hPSC-derived islets, mitochondria

## Abstract

Despite decades of research, there is still no effective cure for type 1 diabetes, except islet transplantation, which is limited by healthy islet donors’ shortage. Transplantation of surrogate insulin-producing β-cells from human pluripotent stem cells (hPSC) may provide an alternative solution when endogenous β-cells are nearly depleted. However, most differentiation protocols generate immature β-cells with impaired glucose-stimulated insulin secretion (GSIS) compared to primary β-cells. During β-cell development, there is a metabolic shift from glycolysis to OXPHOS metabolism to meet the increasing energy demand, suggesting that mitochondrial function is essential for hPSC-derived β-cell differentiation. This study investigated mitochondrial development and function during the differentiation process of hPSC into islet organoids. Two hPSC cell lines (HUES8 and iPSC824) were differentiated into islet organoids using a 6-stage differentiation protocol. Relevant stage-specific markers and GSIS were measured. Mitochondrial biogenesis, dynamics, morphological remodeling, and function were assessed to investigate mitochondria role in β-cell development. Mitochondrial contribution to total ATP production and lactate production were measured to assess the metabolic shift. The results showed, in both cell lines, >96% OCT4^+^ cells (pluripotency marker) at S0, >70% PDX1^+^ cells (pancreatic progenitor marker) induction at S3 and β-cells at S6 evidenced by NKX6.1^+^/insulin^+^ cells. The stage-specific markers measured by flow were confirmed by immunofluorescence, and functionality was assessed by responding to glucose stimulation. Moreover, our results demonstrate that the metabolic shift from glycolysis to OXPHOS occurred at pancreatic progenitor stage as evidenced by an increase in mitochondrial intensity. This shift in mitochondrial intensity coincided with (i-) enhance in mitochondrial biogenesis and dynamics (ii-) mitochondrial morphology remodeling from round shaped to elongated and well-defined cristae (iii-) increase in mitochondrial function related genes, and proteins as well as increased OXPHOS contribution to total ATP production (iv-) downregulation of “disallowed” glycolytic genes and proteins that ultimately promote functional islets.

## 1 Introduction

Type 1 diabetes (T1D) accounting ~10% of diabetic patients, is characterized by a selective autoimmune destruction of pancreatic β-cells leading to nearly complete loss of insulin production that typically develops over several years. The first-line pharmacological treatment plan for T1D predominantly relies on exogenous insulin injections (1). Whilst the extrinsic insulin supplementation is considered as a life-saving treatment, it is associated with an elevated risk of hypoglycemia episodes for many patients (2, 3) and not as efficient as the endogenous pancreatic islets for glycemic control. To overcome these limitations, advanced and emerging treatment strategies are being explored, including islet transplantation (4). Transplantation of cadaveric islets is an effective alternative therapy in restoring long-term glycemic control when endogenous β-cells have already been practically depleted (5, 6). However, this is limited by the critical shortage of healthy islet donors. Human pluripotent stem cells (hPSCs) possess the ability to self-renew and differentiate into various cell types, including β-cells. Thus, generation of hPSC-derived β-cells potentially offer an alternative source of surrogate insulin-producing β-cells. Over the last two decades, many *in vitro* protocols from multiple research groups have been developed to differentiate hPSCs into insulin-producing cells with key features of bona fide mature β-like cells (7–9). However, most of the differentiated hPSC-derived insulin-expressing β-cells exhibit functionally immature phenotypes such as impaired glucose-stimulated insulin secretion (GSIS) or a weaker response to GSIS relative to primary β-cells (10, 11). Fully developed β-cells establish glucose responsiveness through two distinct processes: differentiation and maturation. Since functional maturation of β-cell can be achieved post-implant (12), generating pancreatic progenitors or immature β-cells may be adequate when considering the cost associated with extended culture for the maturation stage. Nevertheless, fully functional hPSC-derived β-cells remain still preferable for diabetes therapy and drug screening. The role of pancreatic β-cell mitochondria in impaired GSIS and diabetes mellitus (DM) is well described (12). Well-developed mitochondrial activity is a cellular component required for GSIS. Both human and rodent fetal and rodent neonatal islets (13–15) display impaired glucose responsiveness despite adequate insulin reserves (16). Comparison of their transcriptomes to adult β-cells showed several differentially expression of key metabolic genes including mitochondrial membrane shuttles (17) and enhanced mitochondrial membrane shuttles has been shown to significantly improve GSIS (18). Also, iPSC-derived β-cells generated *in vitro* are often characterized by low amplitude of GSIS as compared to native pancreatic β-cells in spite of equal amount of mitochondrial mass per cell (19), suggesting the presence of metabolically dysfunctional mitochondria in differentiated hPSC-derived β-cells (19). Stem cells have the unique feature of transitioning between the metabolically distinctive states of quiescence to proliferation. Thus, quiescent stem cells primarily rely on glycolysis for energy production to avoid generation of free radicals (20). Emerging evidence indicates that during β-cell development, there is a metabolic shift from glycolysis to OXPHOS metabolism to meet the increasing energy demand (21) as OXPHOS generates higher amounts of ATP than glycolysis, suggesting that robust mitochondrial maturation is an essential requirement for the differentiation of hPSCs into functional β-cells. More importantly, multiple studies showed that both ESCs and iPSCs (22–25) have spherical mitochondria with poorly developed cristae compared to their long, branched, and cristae-rich somatic cell counterparts. Moreover, the metabolic shifts from glycolysis to OXPHOS in the acquisition of lineage commitment and differentiated state is associated with mitochondrial remodeling (25). Lv and colleagues reported that definitive endoderm (DE) cells had more mature mitochondria and lower lactate content than stem cells, suggesting that mitochondria homeostasis is important as cells exit pluripotency into DE (26). Additionally, report from Kieffer’s group revealed distinct nutrient utilization phenotypes during hESC differentiation showing high lactate production up to pancreatic progenitor stage (stage 4) that reduced at endocrine progenitor stage (stage 5) (8), indicating a metabolic switch from glycolysis to OXPHOS by endocrine progenitor cells. However, the underlying mechanism for the metabolic shift was not studied and only embryonic stem cell (27) lines were used in that study and whether the metabolic shift was cell line specific is unknown as it is widely reported that cell line types substantially affect the differentiation capacity (28). Furthermore, the morphology, distribution, and function of mitochondria have been reported to play a crucial role in the induction of insulin secretion in response to glucose stimulation of differentiated β-cells. Therefore, the aim of this study is to investigate the mitochondrial development and function across the differentiation stages of both human embryonic (HUES8) and induced (iPSC824) pluripotent stem cells into pancreatic islet organoids.

## 2 Material and methods

### 2.1 Stem cell culture and *in vitro* pancreatic β-cell differentiation

Both embryonic (HUES8) and induced (iPSC824) pluripotent stem cell lines were used in this study. iPSC824 line was generated by Qatar Biomedical Research Institute stem cell core from commercially available consented healthy (female) donors’ dermal fibroblasts (29). Undifferentiated HUES8 and iPSC824 cells were cultured on Matrigel-coated (Corning Inc, Bedford, MA, USA) 6-well plates and maintained in mTeSR+ (StemCell Technologies Inc. CAT#100-0276) for expansion. After reaching ~80-90% confluency following at least three passages, cells were dissociated into a single-cell suspension using TrypLE (Gibco; CAT#12604013), counted and seeded at 6 million cells per well of a 6-well plate. Differentiation of stem cells was performed using a 6-stage differentiation protocol as previously published (30). Key small molecules and growth factors added to the differentiation media in each stage of differentiation are as follows: Stage 1 (4 days): 100 ng/mL Activin A, 3µM CHIR99021 (day 1 only) in BE1 media; Stage 2 (2 days): 50 ng/mL KGF in BE2 media; Stage 3 (2 days): 50 ng/ml KGF, 0.25 μM Sant1, 0.2 µM TPPB, 2μM RA, 0.2 µM LDN193189 in BE3 media; Stage 4 (4 days): 50 ng/ml KGF, 0.25 μM Sant1, 0.2 µM TPPB, 0.1 μM RA, 0.2 µM LDN193189 in BE3 media; Stage 5 (7 days): 1 µM Latrunculin A (day 1 of Stage 5 only) 0.25 µM SANT1, 0.1 µM RA, 1 µM XXI, 10 µM Alk5i II and 1 µM T3 in BE5 media; Stage 6 (15 days): ESFM media every other day. At day 8 of stage 6, cells were dissociated from the 6-well plate using TrypLE and reaggregated by transferring to a new 6-well plate and shaken at 70 rpm.

### 2.2 Flow cytometry

At each stage of differentiation, cells were dissociated using TrypLE Express (Gibco, Cat#12604013) at 37 °C to obtain single cells. The cells were washed twice with cold stain buffer (554656) then fixed with 4% PFA (J61899.AP) for 20 minutes at 4 °C. After fixation, they were washed twice with stain buffer (554656) and incubated in permeabilization/washing buffer (51-2091KZ) containing primary antibodies overnight at 4 °C. Subsequently, cells were washed three times by centrifugation (300 rcf, 5 min) using permeabilization/washing buffer and then incubated with secondary antibodies in the same buffer for 2 hours at room temperature. After three additional washes, the cells were resuspended in stain buffer and analyzed using Cytoflex flow cytometer (Beckman Coulter). For mitotracker flow cytometry, TrypLE Express dissociated cells were washed twice with PBS and then incubated in PBS containing Mitotracker green (M46750) for 30 minutes at 37 °C, as per manufacturer’s protocol. The cells were washed twice more with PBS and then analyzed by flow cytometry. The median flow intensity value was used as a proxy measure of mitochondrial intensity. All flow cytometry data were processed with FlowJo software, and the results presented are representative of at least three independent differentiation experiments.

### 2.3 Measurement of mitochondrial ROS

Mitochondria are the primary sites of ROS production during electron transport chain. To measure mtROS production across different stages (S0, S3 and S6) of β-cell differentiation, MitoSox Red mitochondrial superoxide (M36007) staining was used following the manufacturer’s instructions. Cells were stained with 2.5 μM MitoSox Red in pre-warmed buffer (HBSS with Calcium and Magnesium) solution for 30 min at 37 °C and 5% CO_2_. The cells were washed gently three times with warm HBSS buffer and then analyzed by flow cytometry. The median flow intensity value was used as a proxy measure of mtROS.

### 2.4 Immunofluorescence staining

At each stage of the differentiation, cells grown on coverslips were washed, and fixed in 4% PFA for 1 h on ice. Fixed cells were washed with PBS, permeabilized with permeabilization buffer (0.3% Triton-X in PBS) for 30-min and blocked with blocking buffer (6% BSA, 2% donkey serum, 0.3% Triton-X in PBS) for at least 2 hours. They were then incubated with primary antibodies overnight at 4 °C in blocking buffer, followed by three washes and incubation with secondary antibodies for 2 hours at room temperature in the same buffer. After additional washes, the cells were incubated with DAPI for 10-min, washed twice, and mounted with a coverslip using permount mounting media (SP15-100). Also, to determine mitochondrial activity, cells grown on coverslips were stained with 100 nM of MitoTracker Deep Red (M22426) for 30 min, washed and fixed in 4% PFA for 20-min, then washed and incubated with DAPI for 10-min. For co-staining of mitotracker with PDX1, after staining with 100 nM of MitoTracker Deep Red and fixation with 4%PFA, cells were permeabilized, blocked and incubated with PDX1 antibody (1/300) for 2 hours at room temperature, followed by three washes and incubation with secondary antibodies for 2 hours at room temperature. Thereafter, cells were washed and incubated with DAPI for 10-min. In addition, co-localization of mitochondria with lysosomes was performed. Briefly, cells were incubated with 1µM of Lyso Dye (Cat#2690201B) for 30-min following Mitrotracker staining. Images were acquired using a 20 ×/0.8 numerical aperture (NA) objective (LD LCI Plan‐Apochromat; Carl Zeiss Inc., Oberkochen, Germany) using confocal microscopy on a laser scanning microscope (LSM 780; Carl Zeiss Inc., Oberkochen, Germany). Images were analyzed using ZEN imaging software (Carl Zeiss Inc.). Images shown are representative of at least three biologically separate differentiations.

### 2.5 Gene expression using real-time RT-PCR

At each stage of the differentiation (S0, S3, S6), total RNA was extracted from cells using the RNeasy Mini Kit (74104) following the manufacturer’s instructions, then converted to cDNA using High-Capacity cDNA Reverse Transcription Kit (4374966). Gene expression levels were analyzed by RT-PCR on a QuantStudio 6 Flex system using SYBR Green (43 (4309155). Relative expression was determined using the Livak comparative ΔΔCt method (31). GAPDH and Actin genes were used as housekeeping genes. The primers were ordered from Integrated DNA Technologies (IDT Company, Coralville, IA) and their sequences are listed in **Table 1**.

**Table 1:** Primer list and sequences.

Gene	Forward	Reverse
TFAM	5’-TCCCCCTTCAGTTTTGTGTA-3’	5’-GTTTTTGCATCTGGGTTCTG-3’
PGC1α	5’-GCCAAACCAACAACTTTATCTCTTC-3’	5’-CACACTTAAGGTGCGTTCAATAGTC-3’
PPARγ	5’-CGAGGACACCGGAGAGGG-3’	5’-TGTGGTTTAGTGTTGGCTTCTTT-3’
SIRT3	5’-TGTACAGCAACCTCCAGCAG-3’	5’-AAGGGCTTGGGGTTGTGAAA-3’
DRP1	5’-AAGAACCAACCACAGGCAAC-3’	5’-GTTCACGGCATGACCTTTTT-3’
MFN1	5’-TTGGAGCGGAGACTTAGCAT-3’	5’-TTCGATCAAGTTCCGGATTC-3’
MNF2	5’-AGAGGCATCAGTGAGGTGCT-3’	5’-GCAGAACTTTGTCCCAGAGC-3’
OPA1	5’-GGCCAGCAAGATTAGCTACG-3’	5’-ACAATGTCAGGCACAATCCA-3’
NDUSF1	5’-TGCCAAAGGATTGTTTCATT-3’	5’-TGCTGAGCTCTACCCTCAGT-3’
NDUSF2	5’-GTTCCTCCAGGAGCCACATA-3’	5’-CTTGTCCAAACCAGCCAGAT-3’
COX-1	5’-CCCCGCATAAACAACATAAG-3’	5’-CTGTTCAACCTGTTCCTGCT-3’
ATP5A1	5’-GCTCCTTACTCTGGCTGTTCCA-3’	5’-GCGGAGCAACAGAGACATCTGA-3’
GCK	5’-ATGCTGGACGACAGAGCC-3’	5’-CCTTCTTCAGGTCCTCCTCC-3’
GLUT1	5’-ATGGAGCCCAGCAGCAA-3’	5’-GGCATTGATGACTCCAGTGTT-3’
HK1	5’-CGGGACACTCTACAAGCTTC-3’	5’-CTCAGACAGGAGGAAGGACA3’
HK2	5’-ACCATGACCAAGTGCAGAAG-3’	5’-AGCCCTTTCTCCATCTCCTT-3’
PFK	5’-GAGCACCTGACGGAGAAAAT-3’	5’-GCCTTTGCCCTCTTCTGAAT-3’
PGK	5’-CCAGAGGATTAAGGCTGCTG-3’	5’-GAGTACTTGTCAGGCATGGG-3’
LDHA	5’-GCCTTGATTTAGTCCAGCGA-3’	5’-TCCACTCAACTTCCAGGCTA-3’
Actin	5’-TCATGAAGTGTGACGTGGAC-3’	5’-GCAGTGATCTCCTTCTGCAT-3’
GAPDH	5’-CCACTCCTCCACCTTTGACG-3’	5’-ATGAGGTCCACCACCCTGTT-3’

### 2.6 Whole cell lysates and western blot analysis

At different stages (S0, S3, S6) of the differentiation, total proteins were extracted from cells using RIPA buffer (Thermo Fisher Scientific, Cat#89901). Protein concentration was determined by the BCA method using BSA as a standard, and 20 µg of proteins were loaded on 10% SDS-PAGE gels to detect OXPHOS, PGC-1α, TFAM, LDHA, HK2, PDH, PINK1, LC3B. Proteins were then transferred onto PVDF membranes, blocked with 5% non-fat dried milk in Tris-buffered saline containing 0.05% Tween 20 (TBST) for 1 h, and then probed with the primary antibody for overnight at 4 °C. GAPDH was used as internal control. Antibodies recognizing OXPHOS, PGC-1α, TFAM, HK2, LDHA, PDH, PINK1, LC3B and GAPDH were used at dilutions of 1:1000, 1:1000, 1:1000, 1:1000, and 10.000, respectively. After washing, the membranes were incubated with horseradish peroxidase-conjugated secondary antibody at a dilution of 1:2000 for 2 h at room temperature. Protein bands were visualized by chemiluminescence, and the images were captured using the ChemiDoc XRS+ system (Bio-Rad, Hercules, CA).

### 2.7 Glucose stimulated insulin secretion assay

Dynamic GSIS with the PERI5 perfusion system enables sample collection at short intervals (2–5 minutes), providing a time profile of insulin release compared to static GSIS and allowing accurate detection of the biphasic pattern of insulin secretion considered as an important indication of adequate β-cell function (32). However, due to the lack of a perfusion dynamic platform, a static GSIS assay—commonly used to evaluate glucose responsiveness in stem cell–derived islets—was performed (29, 33–37). Thus, both HUES8- and iPSC824-derived islet aggregates (35 days of differentiation) were collected for static GSIS assay. Krebs buffer (KB) was freshly prepared and consists of 128 mM NaCl, 5 mM KCl, 2.7 mM CaCl2, 1.2 mM MgSO4, 1 mM Na2HPO4, 1.2 mM KH2PO4, 5 mM NaHCO3, 10 mM HEPES, 0.1% BSA in deionized water. Cell aggregates were washed twice with low-glucose (2.8 mM) KB and incubated for 1 h in 37 °C incubator. They were washed once in low-glucose KB to remove any residual insulin, incubated again in low-glucose KB for 1 h, and the supernatant was collected. Then to challenge the cells, aggregates were incubated in high-glucose (20 mM) KB for 1 h, and the supernatant was collected. Finally, aggregates were incubated in low-glucose KB containing 30 mM KCl (forced depolarization challenge) for 1 h, and then the supernatant was collected. Aggregates were finally dispersed into single cells using TrypLE Express, and cell number was counted automatically to normalize C-peptide level by the cell number to avoid potential bias introduced by variations in islet organoid size between independent experimental replicates. Supernatant samples containing secreted C-peptide (a proxy marker for insulin co-released at equivalent molar) were processed using the human ultrasensitive C-peptide ELISA kit (10-1141-01).

### 2.8 ATP and lactate assays

Lactate and intracellular ATP levels were measured using the Glycolysis/OXPHOS assay kit (G270) as per manufacturer’s protocol. Total cellular ATP is the result of glycolytic and mitochondrial ATP production. For the contribution of mitochondrial ATP production to the total cellular ATP at different stages of differentiation, 100,000 cells from S0, S3 and S6 were seeded in 96-well plate with a stage corresponding medium supplemented without or with 2.5 µmol oligomycin (a specific inhibitor of mitochondrial ATP synthase (i.e. OXPHOS pathway) and incubated for 3 hours. Then, 100 µl of ATP assay buffer was added into each well followed by reverse pipetting and mixing in an orbital shaker for 2 min. The plate was incubated for 10 min at room temperature and then, the luminescence was measured. For lactate production, spent media was collected at S0, S3 and S6, centrifuged and frozen at −80 °C until being assayed. Lactate was measured following the manufacturer’s instruction. Lactate production rate was calculated using the following equation (8):

$Q_{lac}=\frac{S_{f}-S_{0}}{(N/V_{f})\times \Delta t}$ refers to lactate production rate. *Sf* and *S*_0_ are the concentrations of lactate in the spent and fresh media, respectively. *N* and *Vf* refer to the cell number and the media volume at the time of collection, respectively. Δ*t* is the time between media change.

### 2.9 Transmission electron microscopy

For transmission electron microscopy, S0, S3 and S6-derived cells were washed with PBS and fixed in 2.5% glutaraldehyde, 2% PFA overnight at 4 °C as previously described (38). Briefly, after fixation, cells were washed 3 times with PBS and embedded in 4% agarose. Cells were then incubated in 2% osmium-tetroxide solution for 30-min on ice followed by incubation in 1% thiocarbohydrazide solution for 20-min on ice. Samples were again incubated in 2% osmium-tetroxide solution for 30-min on ice followed by 2% uracyl acetate solution overnight on ice. After 5 times washes with distilled water, samples were dehydrated with a graded series of ethanol (30%, 50%, 70%, 96%, 100%), and then infiltrated with 100% propylene oxide solution for 15 min followed by incubating samples in 2:1 solution of propylene oxide: resin for 60 min on a rocker under constant agitation, and in 1:1 solution of propylene oxide: resin for 60 min, then in 1:2 solution of propylene oxide: resin for 90 min and finally in 100% resin embedding media overnight on a rocker under constant agitation. The samples were again embedded in resin for 5 hours at room temperature on a rocker with open lid and then transferred to the mold and incubated in an oven at 65 °C for 48 hours. Afterwards, blocks were cut into 100 nm thickness using an ultramicrotome and post-stained with uranyl acetate for 10 min and then proceed to TEM imaging.

### 2.10 Statistical analysis

All assays were performed at least in triplicate and a minimum of three independent experiments. Results are presented as means ± SEM and were plotted using GraphPad (Prism v7, La Jolla, CA). We used one-way ANOVA for comparison of the groups with *post-hoc* Tukey’s test or the student t test and paired two-sided t-test, as appropriate. A p-value < 0.05 was considered statistically significant.

## 3 Results:

### 3.1 Differentiation of hPSC lines into islet organoids with functional β-cells

**Figure 1:**
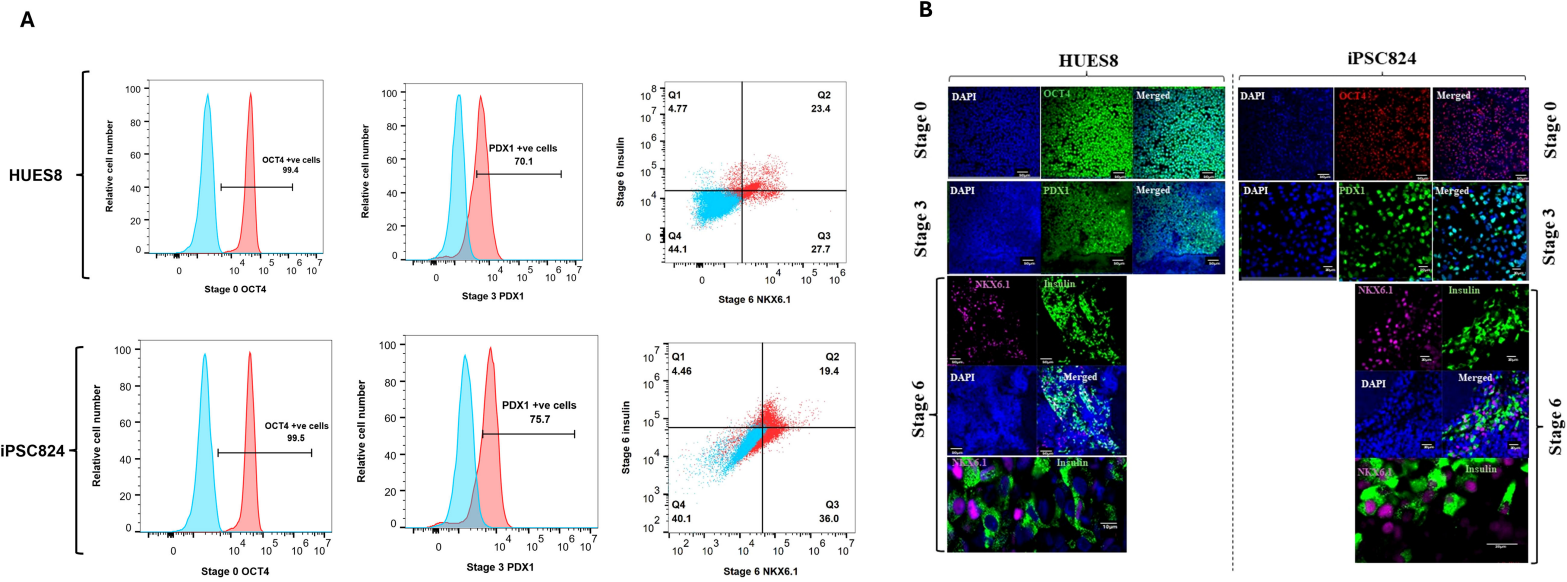
Characterization of stage-specific marker expression during β-cell differentiation. Representative flow cytometry plots of relevant stage-specific markers of HUES8 and iPSC824 differentiation into insulin-expressing β-cells **(A)**. Cells were collected at different stages of differentiation including stage 0 (OCT4 for pluripotency), stage 3 (PDX1 for pancreatic progenitor) and stage 6 (NKX6.1/insulin for β-like cells). Data were analyzed using FlowJo and flow cytometry plots are representative of 3 independent experiments. Representative immunofluorescence images showing the expression of key developmental markers in HUES8 and iPSC824 cell lines at various stages of the differentiation **(B)**. Cells were stained for DAPI (blue), OCT4 (green/red) at the pluripotency stage, PDX1 (56) at the pancreatic progenitor stage, and NKX6.1 (purple) and Insulin (56) at the β-cells stage. The images were acquired using a 20X or 40X magnification with a Zeiss LSM 780 confocal microscope (Carl Zeiss, Oberkochen, Germany). Scale bar = 10 µm, 20µm or 50µm. Images are representative of 5 independent experiments.

**Figure 2:**
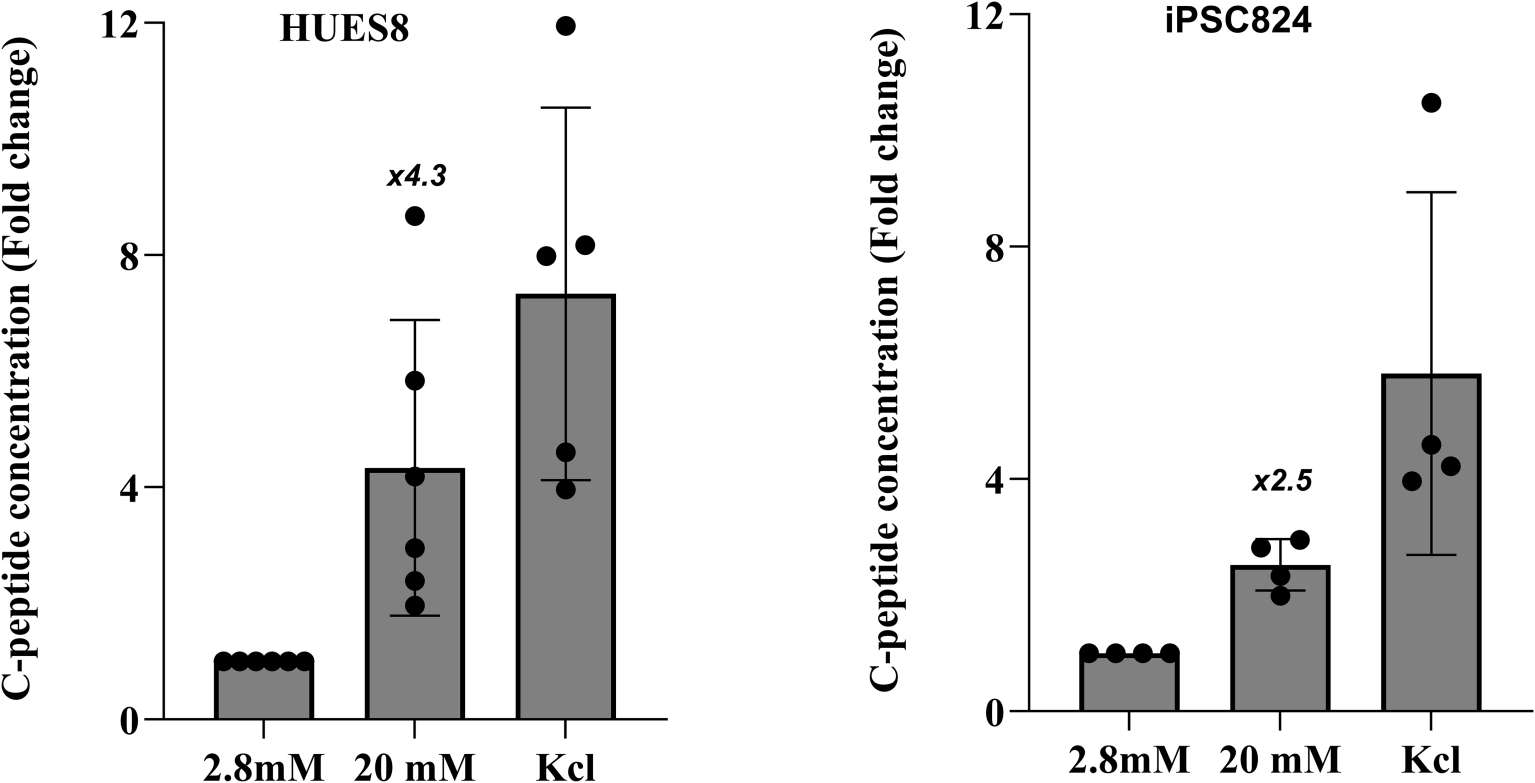
Functional characterization of Stage 6 hPSC-derived islets via GSIS. Static glucose-stimulated insulin secretion (GSIS) in stage 6 islets derived from HUES8 (n=5 independent biological replicates) and iPSC824 (n=4 independent biological replicates) cell lines. Islets were sequentially incubated for 1 hour with 2.8 mM (low) and 20 mM (high) glucose and then with 30mM KCl. C-peptide concentration were measured by ELISA kit and normalized to cell number. A paired two-sided t-test was used to compare c-peptide concentration under low and high glucose conditions within each cell line.

Using a 2D differentiation protocol (30), both HUES8 and iPSC824 stem cell lines were differentiated into insulin-expressing cells. The iPSC824, home generated iPSC expressed pluripotency markers SOX2, OCT4, SSEA4, and TRA1-60; and successfully differentiated into ectoderm, mesoderm, and endoderm germ layers; and had normal 46, XX karyotype (29). At each stage of the differentiation, stage-specific markers were assessed to determine the efficiency of the differentiation using flow cytometry and immunofluorescence staining. The results showed >96% OCT4^+^ cells (pluripotency marker) at S0, >70% PDX1^+^ cells (pancreatic progenitor marker) induction at S3 in both cell lines. Subsequently, β-cell-specific markers were induced, as evidenced by the generation of 23.4% and 19.4% NKX6.1/insulin double-positive cells in HUES8 and iPSC824, respectively at S6 (**Figure 1A**). The expression of stage-specific markers was also confirmed by immunofluorescence (**Figure 1B**). Additionally, to assess the functionality of hPSC-derived islets, static GSIS assay was performed. Data displayed in **Figure 2** indicated that, in HUES8, a 4.3-fold increase in C-peptide secretion was observed in stage 6 derived clusters when challenged from 2.8 mM (low) to 20 mM (high) glucose, suggesting a glucose-responsive (i.e. functional) pancreatic β-cells. similar results were observed in iPSC824 with a 2.5-fold increase in C-peptide secretion when challenged with high glucose (**Figure 2**). Taken together, our data indicate that both HUES8- and iPSC824-derived islets displayed a consensual functional phenotype despite of a lower stimulation index (a lower insulin secretion following high glucose challenge) observed in iPSC824 as compared to HUES8.

### 3.2 Mitochondrial biogenesis and remodeling upon differentiation of hPSC

**Figure 3:**
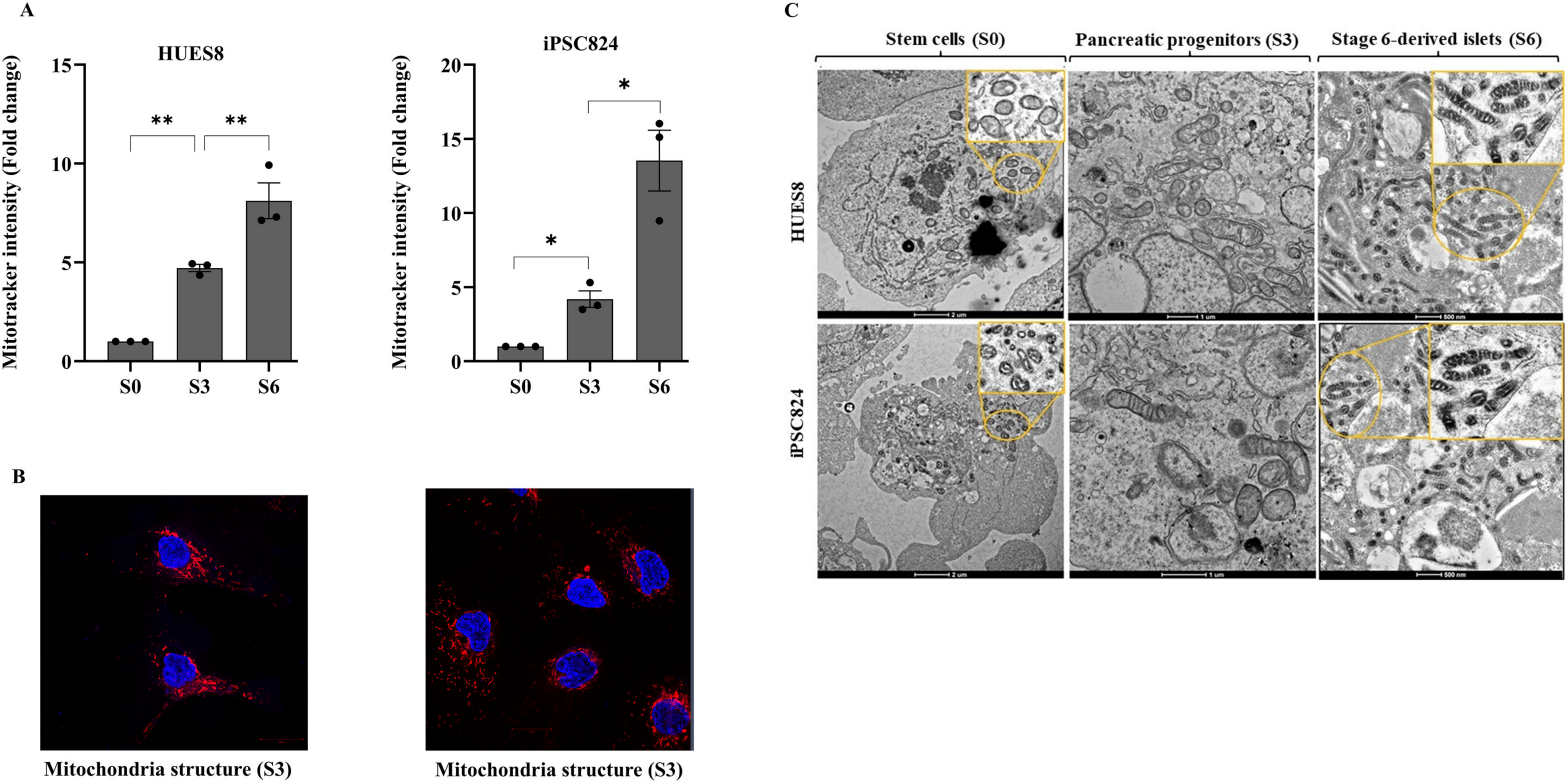
Mitochondrial intensity and stage-specific morphological and ultrastructural remodeling of mitochondria during β-cells differentiation. **(A)** Mitochondrial intensity (a proxy measure of mitochondria activity) using Mitotracker green (M46750) across different stages (S0, S3 and S6) of β-cell differentiation. **(B)** Representative immunofluorescence images showing active mitochondrial structure using MitoTracker Deep Red (Cat no:M22426) at stage 3 in both HUES8 and iPSC824. **(C)** Representative TEM images depicting ultrastructure of mitochondria in both HUES8 and iPSC824 at stage 0, stage 3 and stage 6. *P < 0.05; **P < 0.01.

**Figure 4:**
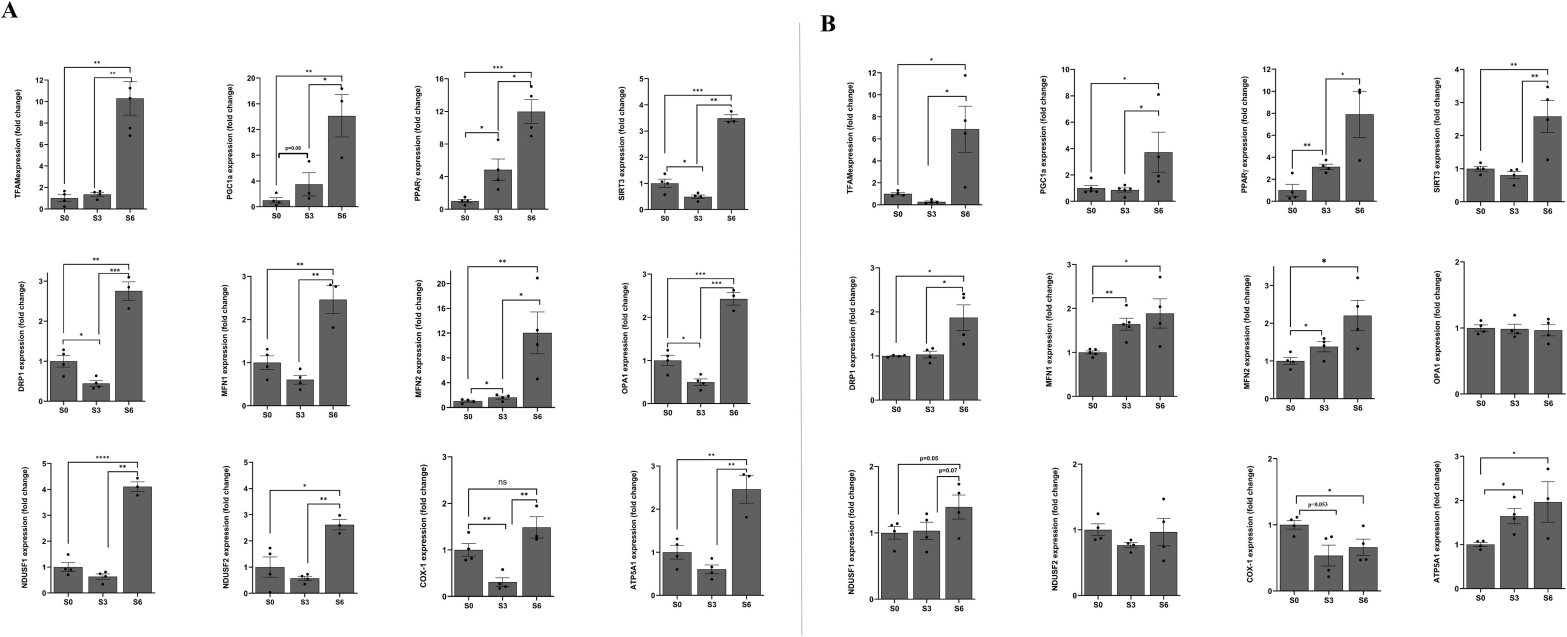
Upregulation of mitochondrial biogenesis and bioenergetic genes during *β*-cells differentiation. Relative mRNA expression of mitochondrial biogenesis (TFAM, PGC1a, PPARγ), dynamic (DRP1, MFN1, MFN2, OPA1) and regulation (SIRT3), and OXPHOS (NDUSF1, NDUSF2, COX1, ATPSA1) genes at stages 0, 3, and 6 of HUES8 **(A)** and iPSC824 **(B)** differentiation. Relative expression was calculated using the comparative ΔΔCT method, and fold change (2^−ΔΔCt^) was determined. Data are represented as mean ± SEM. (n=4 for both HUES8 and iPSC824). One-way ANOVA for comparison of the groups with *post-hoc* Tukey’s test was used. A P-value < 0.05 was considered statistically significant. *P < 0.05; **P < 0.01; ***P < 0.001.

**Figure 5:**
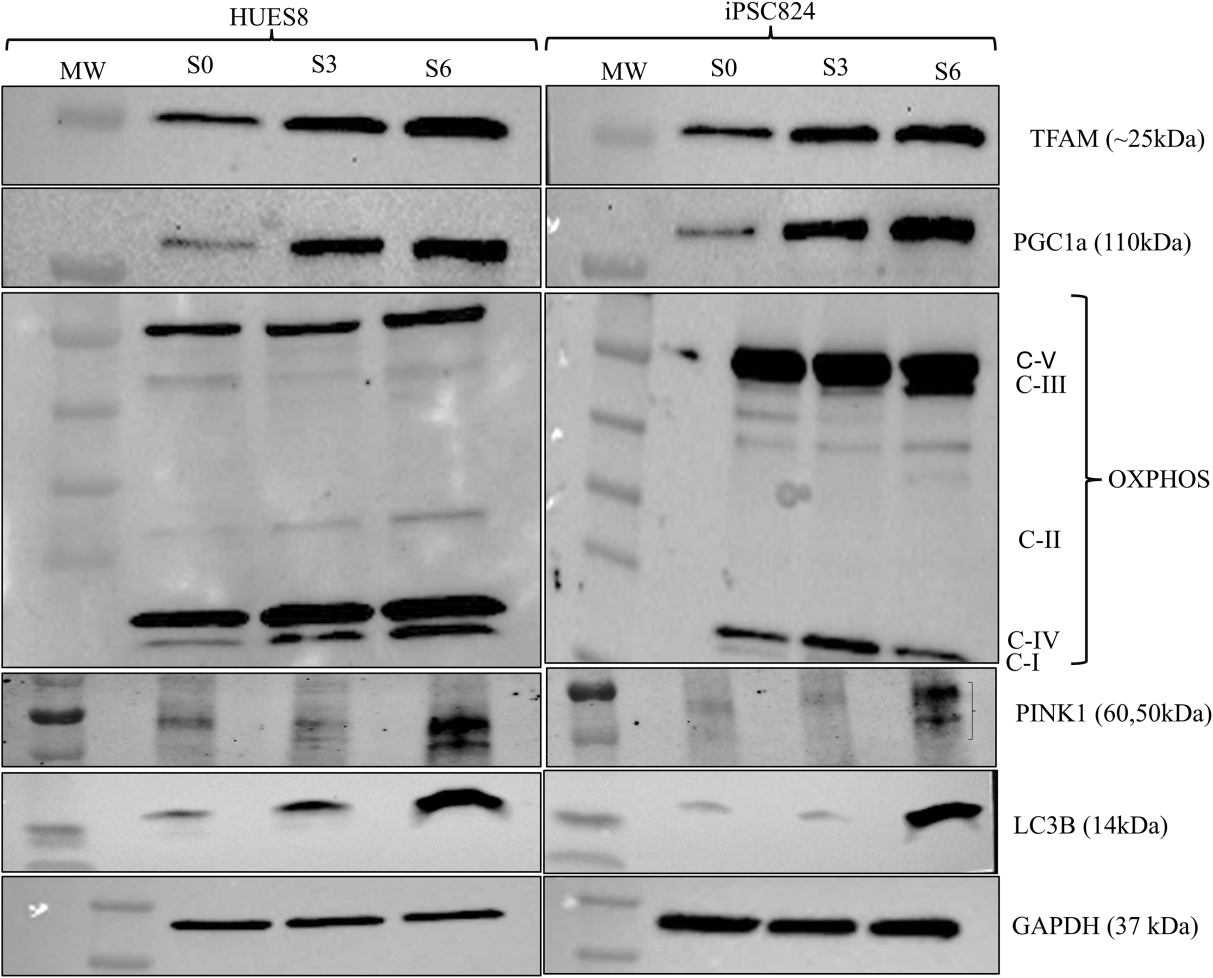
Expression of proteins involved in mitochondrial biogenesis, OXPHOS and mitophagy. Representative western blots showing the expression of mitochondrial proteins involved in biogenesis (TFAM, PGC1α), oxidative phosphorylation (OXPHOS) and mitophagy (PINK1 and LC3B) measured at S0, S3 and S6 in both HUES8 and iPSC824 cell lines. GAPDH was used as internal control to monitor protein loading differences. Full-length blots are displayed in **Supplementary figure**.

**Figure 6:**
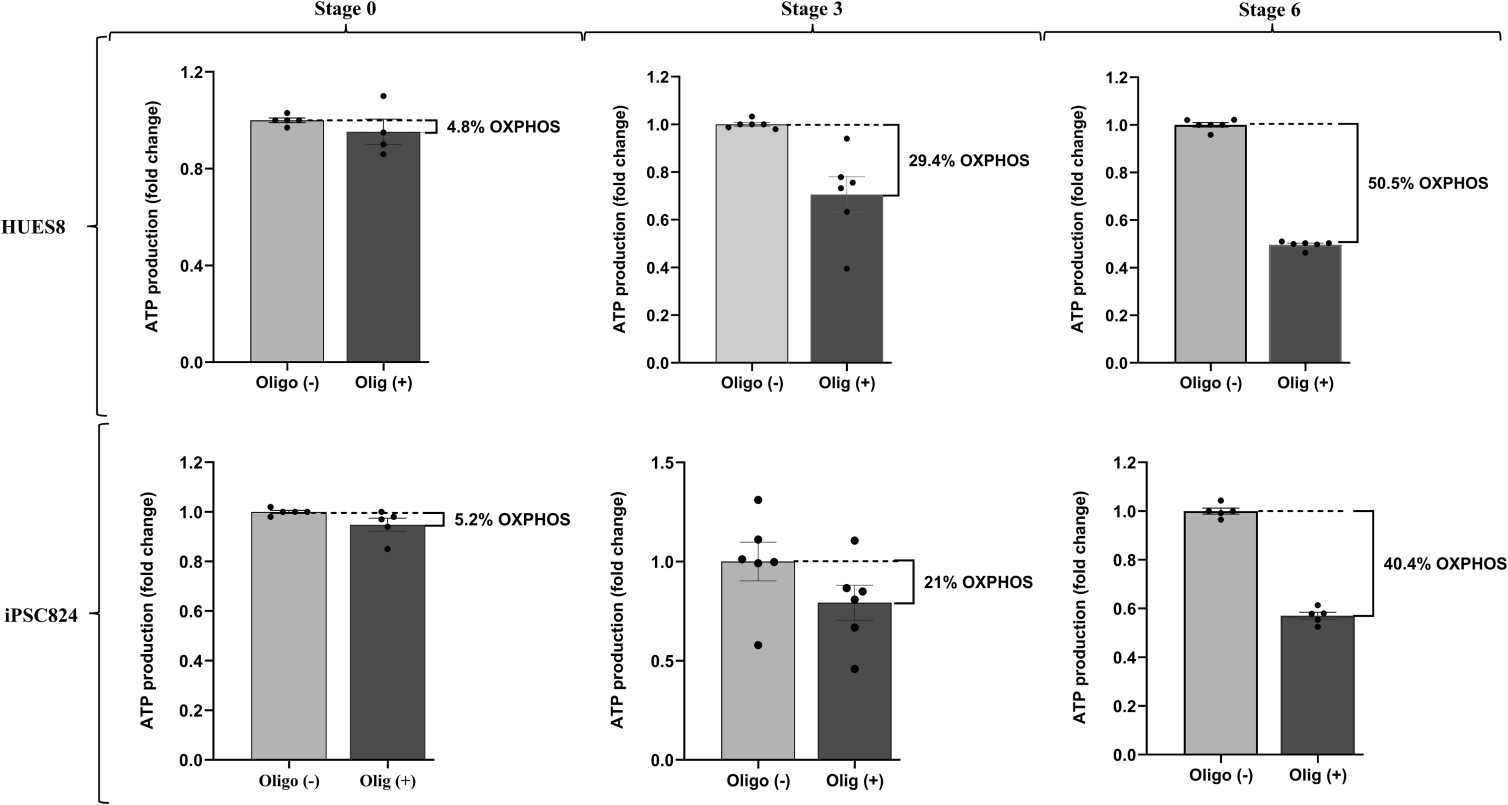
Metabolic transition from glycolysis to oxidative phosphorylation (OXPHOS) during *β*-cell differentiation. Mitochondrial OXPHOS contribution to total ATP production across differentiation stages (S0, S3 and S6) in HUES8 and iPSC824; n=5–6 per cell line.

During pancreatic β-cell development, a metabolic switch occurs, transitioning the cells from a low-energy (glycolysis-dependent) to a high-energy producing (OXPHOS-dependent) state. To understand the underlaying mechanism for this metabolic shift, we studied mitochondrial biogenesis, dynamics, and functional remodelling during pancreatic β-cell development. To assess mitochondrial biogenesis and dynamics, we first measured mitochondrial intensity using Mitotracker green and flow cytometry at different stages of the differentiation (**Supplementary Figure 1**). Interestingly, the biggest shift in mitochondrial intensity occurs at stage 3 of the differentiation, representing the development of pancreatic progenitors. The data displayed in **Figure 3A** showed a 4.7-fold and 4.2-fold increase in mitochondria intensity in HUES8 (p<0.01) and iPSC824 (p<0.05) in stage 3 (pancreatic progenitors) as compared to stage 0 (stem cells) respectively, indicating a substantial increase in mitochondria activity. The mitochondrial intensity further increased at stage 6 (β-cell organoids) in both cell lines (**Figure 3A**) by an 8.1-fold and 13.5-fold increase in HUES8 (p<0.001) and iPSC824 (p<0.001), respectively. The importance of mitochondrial in mediating stem cell fate is increasingly admitted (25). Thus, to investigate the changes in mitochondrial biogenesis and dynamics during the differentiation process, we measured and compared the expression of key representative genes involved in mitochondrial biogenesis (TFAM, PGC1α, PPARγ), fission (DRP1), and fusion (MFN1, MFN2, and OPA1) at S0, S3, and S6 of the differentiation using qPCR; and data are displayed in **Figure 4**. In HUES8-derived cells, when compared with S0, S3 cells exhibited a 1.35-fold, 3.5-fold and 4.8-fold increases in TFAM (p=0.37), PGC1α (p=0.08), and PPARγ (p<0.05) expression, respectively. In iPSC824-derived cells, S3 cells exhibited a 3.1-fold increase in PPARγ (p<0.05) expression when compared to S0. At S6, expression of genes regulating mitochondrial biogenesis (TFAM, PGC1α, PPARγ), fission (DRP1), and fusion (MFN1, MFN2, and OPA1) was all significantly upregulated relative to S0 and S3 (**Figure 4A**), supporting the metabolic shift required during β-cell differentiation. In iPSC824, TFAM and PGC1α were similarly expressed in both S0 and S3, while the expression of PPARγ, MFN1 and MFN2 was significantly upregulated at S3 (p<0.05) as compared with S0. At S6, all mitochondrial-associated genes measured were significantly upregulated (p<0.05) except for OPA1 that showed no statistical change across stages (**Figure 4B**). SIRT3, which indirectly activates the expression of PGC1α was upregulated by 3.5-fold (p<0.001) and 2.6-fold (p<0.01) in S6-derived from HUES8 and iPSC824, respectively (**Figure 4**). Consistently, the expression of the protein levels of TFAM and PGC1α followed the mRNA pattern (**Figure 5**). Mitochondrial biogenesis and mitophagy tightly cooperate to maintain mitochondrial quality. Accordingly, the upregulation of mitochondrial biogenesis markers at stages 3 and 6 was accompanied by elevated levels of mitophagy-related proteins, including Pink1 and LC3B (**Figure 5**). To complement these data, we examined the morphology of mitochondria by confocal microscopy using both MitoTracker Deep Red staining kit and transmission electron microscopy (**Figures 3B, C**). Representative images showed a remodelling of mitochondrial morphology (maturation) from rounded shape to elongated and well-defined cristae at S3 and S6 as compared to S0 (**Figure 3C**). Interestingly, co-staining data of mitotracker and PDX1, a key transcription factor marker of pancreatic progenitor cells) showed that elongated mitochondrial morphology, indicative of enhanced mitochondrial maturation, is predominantly present in PDX1-positive cells than in PDX1-negative cells (**Supplementary Figure 2**). To examine the shift in metabolic state from glycolysis to OXPHOS as cells differentiate, we blocked the OXPHOS pathway using 2.5 µmol oligomycin, a specific inhibitor of mitochondrial ATP synthase, at different stages of differentiation (S0, S3 and S6) and measured total ATP production. The blockage of OXPHOS pathway resulted in reduction of cellular ATP levels by 50% and 40% at S6 in HUES8 and iPSC824, respectively compared to only ~5% at S0 in both cell lines and 29% (in HUES8) and 21% (in iPSC824) at S3. These data indicate an increase in mitochondrial contribution to total ATP production as cells differentiate (**Figure 6**). This increase in mitochondrial ATP production coincides with significant upregulation in the expression of the following OXPHOS-related genes (NDUSF1, NDUSF2, ATP5A1) (**Figure 4**) and a gradual increase at a protein level in complexes I, II and IV (**Figure 5**) in HUES8-derived cells. Mitochondria are the primary source of cellular reactive oxygen species (ROS), specifically superoxide (O^-2^) generated during OXPHOS. Consistent with this, the sharp rise in mitochondrial intensity observed at stages 3 and 6 led to significantly higher mtROS production compared to stage 0 (**Supplementary Figure 3**). These data highlight the essential that mitochondria maturation plays during hPSC-derived islet differentiation, evidenced by both morphological elongation and a rise in ROS production.

### 3.3 Metabolic transition towards OXPHOS driven by the silencing of “disallowed” glycolytic genes and establishment of beta-cell-specific glycolysis

**Figure 7:**
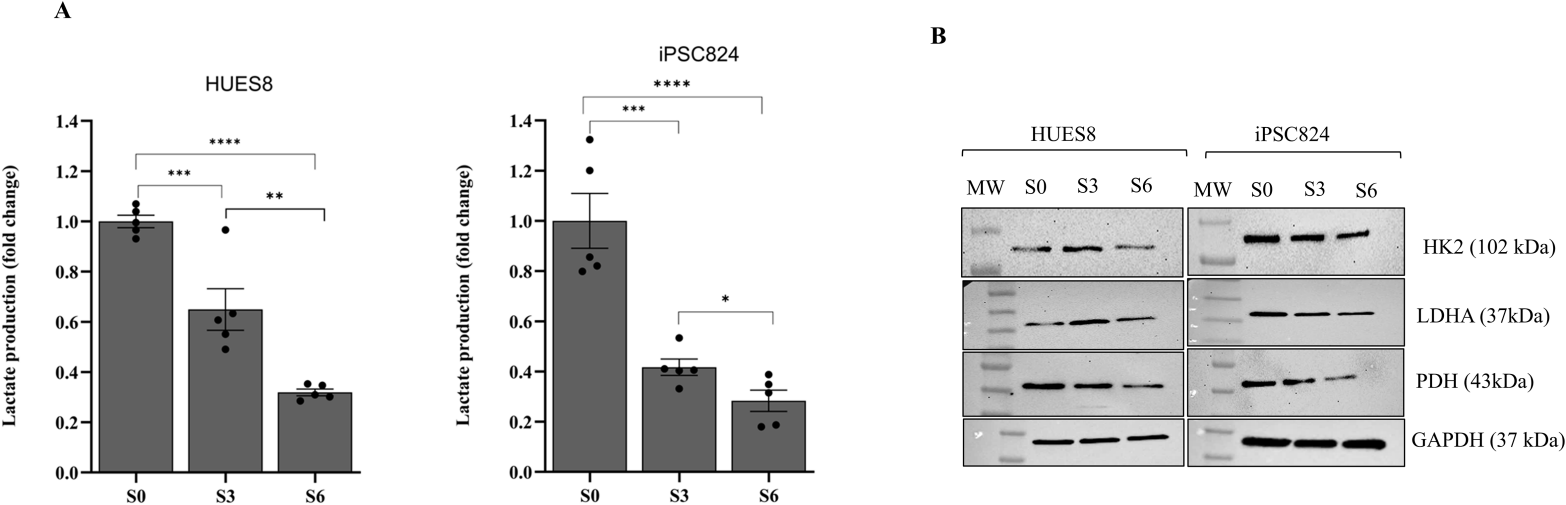
Reduction of glycolytic activity during *β*-cells differentiation. **(A)** Lactate production across differentiation stages (S0, S3 and S6) in HUES8 and iPSC824; n=5 per cell line. **(B)** Representative western blots showing the expression of glycolytic proteins (HK2, PDH, LDHA) measured at S0, S3 and S6 in both HUES8 and iPSC824 cell lines. GAPDH was used as internal control to monitor protein loading differences. Full-length blots are displayed in **Supplementary figure**. One-way ANOVA for comparison of the groups with *post-hoc* Tukey’s test was used. A P-value < 0.05 was considered statistically significant. *P < 0.05; **P < 0.01; ***P < 0.001; ****P < 0.001.

**Figure 8:**
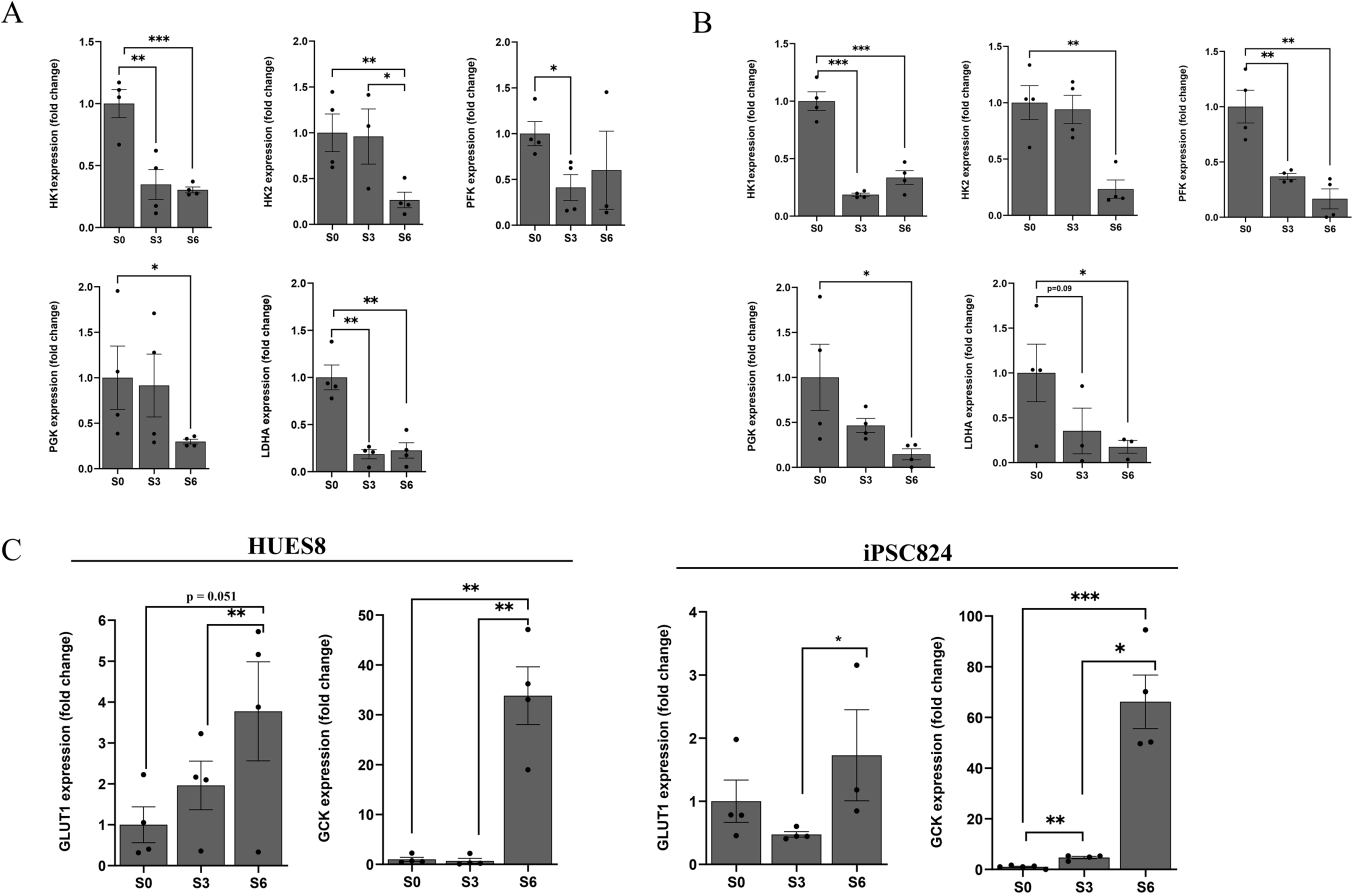
Downregulation of glycolysis genes during *β*-cells differentiation. Relative mRNA expression of glycolytic genes at different stages (S0, S3, S6) of HUES8 **(A)** and iPSC824 **(B)** differentiation. **(C)** GLUT1 and GCK mRNA expression across differentiation stages (S0, S3 and S6) in HUES8 and iPSC824. Relative expression was calculated using the comparative ΔΔCT method, and fold change (2^−ΔΔCt^) were determined. Data are mean ± SEM. (n=4 for HUES8 and iPSC824). One-way ANOVA for comparison of the groups with *post-hoc* Tukey’s test was used. A P-value < 0.05 was considered statistically significant. *P < 0.05; **P < 0.01; ***P < 0.001.

During β-cell development, the metabolic shift towards OXPHOS is accompanied by reduction in lactate production. Lactate production was measured and found to be reduced by 35% and 68% in cells at S3 and S6, respectively when compared with S0 cells in HUES8. Also, in iPSC824, lactate production was reduced by 58% and 72% in cells at S3 and S6, respectively when compared with S0 cells (**Figure 7A**). In this study, we examined whether this metabolic shift is accompanied with repression of the “disallowed” glycolytic genes to support the shift in energy metabolism as hPSCs differentiate into islet organoids. Our results indicated that mRNA expression of the following glycolysis rate-limiting enzymes (HK1 and PFK) were significantly downregulated in S3 (pancreatic progenitor cells) as compared to S0 (stem cells) for both HUES8 and iPSC824 (**Figures 8A, B**). In addition, LDHA that catalyzes pyruvate to lactate during anaerobic glycolysis was also downregulated (**Figures 8A, B**). These genes were further downregulated at stage 6-derived islets in both cell lines (**Figures 8A, B**). Consistent findings were found at the protein level. The expressions of HK2, LDHA, and PDH were reduced upon differentiation (**Figure 7B**). In contrast, the mRNA expression levels of the high-affinity GLUT1 transporter and the rate-limiting enzyme glucokinase (GCK) involved in insulin secretion were significantly upregulated at stage 6-derived islets as compared to stage 0 and stage 3 cells (**Figure 8C**). Together, all these data demonstrated a metabolic shift from glycolysis to OXPHOS during HUES8- and iPSC824-derived islet differentiation.

## 4 Discussion

The importance of mitochondria for β-cell function is well known (39) and functionally mature stem cell-derived β-cell organoids show great promise as an alternative and renewable source for cell therapy for individuals with T1D (40, 41). Despite an improvement in the efficiency of differentiation protocols, hPSC-derived β-like cells generated *in vitro* exhibit suboptimal glucose-stimulated insulin secretion (GSIS) when compared with primary human islets (9), suggesting the generation of immature organoids that may resemble fetal, not adult β-cells (42, 43). Among the various mechanisms underlying this metabolically immature glucose-responsiveness phenotype, mitochondria act as key contributors. Recently, a study showed a gradual etabolic shift from glycolysis, during early stages of β-cell differentiation to oxidative phosphorylation later in the differentiation (8). However, the mechanism through which this metabolic shift occurs remains poorly understood. In the present study, using a stepwise differentiation protocol (30), we demonstrated that the metabolic shift occurs at the pancreatic progenitor (PP) stage (i.e stage 3) of the differentiation as mitochondria biogenesis and remodeling start to take place and this is evidenced by 1. increased both mitochondrial biogenesis (driven by upregulation of key mitochondrial biogenesis regulators, TFAM and PGC1α) and mitochondrial activity 2. mitochondrial morphological maturation from rounded shapes to elongated mitochondria with well-developed cristae, 3. a shift in mitochondrial dynamics toward fusion, via the upregulation of fusion markers (MFN1/2) and the downregulation of fission (DRP1) marker, 4. increased ATP production generated from OXPHOS, 5.- downregulation of glycolytic “disallowed” genes, alongside a reduction in lactate production and its associated proteins (e.g. PDH). Altogether, our data provide compelling evidence of a metabolic shift from glycolysis to OXPHOS, modulated via mitochondrial biogenesis, remodeling, and maturation starting at PP stage. This represents a prerequisite phase that leads to the induction of endocrine precursors and, ultimately, glucose-responsive (i.e. functional) β-cells.

One notable finding from our study is that the metabolic transition appears to initiate at stage 3 of the differentiation, corresponding to the generation of PP cells. This was evidenced by an increase in Mito-Tracker fluorescence intensity at this stage, suggesting elevated mitochondrial activity. The increase in mitochondrial intensity from stage 0 to 3 coincided with a significant increase in PGC1α and TFAM protein expression. This indicates an increase in mitochondria biogenesis to accommodate the shift in energy metabolism from glycolysis to OXPHOS. PGC1α, master regulatory protein for mitochondrial biogenesis, increases the expression of TFAM through activation of nuclear respiratory factors (NRF-1 and NRF-2). TFAM then translocates to the mitochondria to initiate the replication and transcription of mtDNA. As cells develop further, new mitochondria are expected to be generated with functionality that is more tightly coupled with OXPHOS. Our data showed a significant increase in mRNA expression of both PGC1α and SIRT3 from stage 3 to 6 (the β-cell formation stage). SIRT3, known for its role in enhancing PGC1α activity and expression through AMPK and CREB signaling pathway, thereby reinforcing mitochondrial biogenesis as differentiation proceeds to stage 6. Additionally, SIRT3 plays a protective role by increasing the expression of antioxidative enzymes, such as SOD2 and catalase, further supporting mitochondrial functionality during this transition.

The role of mitochondrial dynamics in the process of *in vitro* differentiation has been reported previously. Studies have shown that the activation of mitochondrial fusion in iPSC promotes their differentiation into cardiac cells (44), while the inhibition of dynamic-related protein 1 (DRP1), the master regulator of mitochondrial fission, enhances differentiation into cardiac mesoderm (45). Consistent with these findings, our results showed a downregulation of DRP1 mRNA at the pancreatic progenitor stage (stage 3) when compared with stage 0 (stem cells) in HUES8, alongside an upregulation of MFN2 mRNA (involved in mitochondrial fusion) in both cell lines. This significant upregulation of MFN1 and MFN2 mRNA expression in hiPSC-derived PP cells (stage 3) suggests an increase in mitochondrial dynamics, contributing to the metabolic shift observed at this stage of differentiation. Nevertheless, genetic or pharmacological approaches targeting MFNs would be necessary to confirm the direct role of mitochondrial fusion in the observed metabolic shift. The increase in mitochondrial dynamics is consistent with changes noted in TEM images, where mitochondrial morphology transitioned from round-shaped in stage 0 to elongated, cristae-dense networks by stage 3, indicative of mitochondrial remodeling. As cells loss stemness and transition toward a differentiated state, mitochondria mature, allowing the cells to undergo a metabolic shift from glycolysis to OXPHOS (46–48). To examine mitochondrial contribution to total ATP production, OXPHOS pathway was blocked using oligomycin and ATP production was measured during different stages of differentiation. Oligomycin is a chemical compound that blocks proton conductance through the mitochondrial electron transport chain (ETC) to uncouple OXPHOS, thus inhibiting ATP synthesis (27). This treatment dropped cellular ATP levels by 29% and 21% at S3 in HUES8 and iPSC824, respectively, compared to only ~ 5% at S0, indicating an increase in mitochondrial contribution to total ATP production at the PP stage. PDX1 is a master transcription factor controlling PP development and decreased expression of PDX1 is a hallmark of low PP induction during *in vitro* stem cell differentiation. The role of mitochondria in regulating PDX1 expression has been demonstrated in mice. Specifically, the knockout of family with sequence similarity 3 (FAM3) member A (FAM3A; a mitochondrial protein that enhances ATP production) resulted in reduced PDX1 expression in islets (49). Conversely, FAM3A overexpression upregulated PDX1 expression levels (49), suggesting that mitochondria might regulate PP development by modulating PDX1 expression. FAM3A is also known as a target gene of PPARγ (50); thus the higher expression of PPARγ mRNA observed at stage 3 relative to stage 0 in both cell lines could suggest an indirect effect of PPARγ in mediating PP induction. Interestingly, the increased expression of mitochondrial biogenesis proteins (TFAM, PGC1α) at the PP stage (S3) when compared to stage 0 (stem cell) supports the hypothesis that mitochondria play a role in modulating PDX1 expression to promote PP development.

To determine whether the increase in OXPHOS-derived ATP at PP stage is concomitant with reduced glycolysis, we measured lactate production and found significant decrease in its production at S3 when compared to stem cells (S0); suggesting a reduced reliance on glycolysis by PP as the primary energetic pathway compared with S0 cells (stem cells) that predominantly rely on glycolysis. Consistent with our findings, Lv et al. found that definitive endoderm cells had lower lactate content associated with increased mitochondrial network than stem cells using hESC lines (UC01 and UC06) (26). While OXPHOS generates higher amounts of ATP than glycolysis, differentiated cells require OXPHOS to cope with their high energy needs. For example, PP cells and terminally differentiated β-cells have distinct energy needs. Fully developed and mature β-cells require a significant amount of energy for efficient insulin production and secretion (51); suggesting that β-cells would rely more on OXPHOS as compared with PP cells. Consistent with this, our results showed that OXPHOS contribution to total ATP production in stage 6-derived islets as compared with stage 3-derived PP cells increased from 21% to 40% in iPSC824 and from 29% to 50% in HUES8, revealing the energy need difference between PP and islet cells. Mitochondrial activity, ROS and mitophagy are tightly coordinated processes that ensure mitochondrial quality (52). Accordingly, the increased OXPHOS-induced cellular ATP production observed at stages 3 and 6 coincided with higher levels of mtROS (superoxide (O^-2^)) and mitophagy-related proteins, including Pink1 and LC3B, suggesting that mtROS and mitophagy are essential for mitochondrial remodeling during the hPSC-derived islet differentiation corroborating previous report demonstrating that elevated mROS levels modulate and promote differentiation (53). Acquisition of glucose-responsive mitochondrial oxidative metabolism is one of the key features of functional and mature β-cells. However, newly differentiated β-cells are often not glucose responsive. Functional maturation transforms them into fully competent cells by upregulating islet-enriched transcription factors such as *MAFA, NKX6.1*, which activate glucose-sensing metabolic program and couple it to insulin secretory machinery by promoting mitochondrial OXPHOS (54). The lack of this glucose-responsiveness feature in β-cells under acute inhibition of mitochondrial metabolism (55) demonstrates that OXPHOS is essential for β-cell functionality (39, 56). In the present study, stage 6-derived islets in both hPSC cell lines showed glucose-responsive (i.e. functional) phenotype (increased GSIS in response to high glucose). Activation of mitochondria function; the master regulator controlling the coupling of glucose metabolism to insulin exocytosis is the well described mechanism for GSIS. Also, mitochondria regulate β-cell function via its interaction with NKX6.1, a master transcription factor for β-cell identity and function (57, 58). In this study we observed that glucose-responsive stage 6-derived islets exhibited increased mRNA levels of genes involved in mitochondrial biogenesis (PGC-1α, TFAM), dynamics (DRP1, MFN1, MFN2, OPA1) and oxidative phosphorylation machinery (ATP5A1, NDUSF1, NDUSF2). ATP production via OXPHOS is required for the K_ATP_ channel-dependent pathway to trigger insulin exocytosis (39). Interestingly, mitochondrial dynamics have recently been shown to play an important role in β-cell function. The dynamic processes of mitochondrial fusion and fission are under the control of specific mitochondrial membrane anchor proteins (MFN1 and MFN2, OPA1, DRP1). MFN1 and MFN2 were identified to promote mitochondrial fitness to fuel GSIS in β-cells (59) as impairment of mitochondrial fusion via β-cell specific inactivation of MFN1 and MFN2 resulted in reduced insulin secretion (59). The high expression of MFN1 and MFN2 in stage 6-derived islets confirm their role in β-cell function. Another striking feature of stem cell-derived islets is the reduction of glycolytic genes, most known as “disallowed” genes. Interestingly, inhibition of glycolytic genes has been demonstrated to be required for pancreatic β-cells to achieve and maintain functional maturity. Thus, our results showed that the high expression of mitochondrial genes observed in stage 6-derived functional islets was concomitant with a lower expression of glycolytic genes such as LDHA, PFK, PGK, HK1/2 in both cell lines. Human stem cells primarily use the low-affinity GLUT2 and the rate-limiting glycolytic enzymes HK1/2, while terminally differentiated β-cells rely predominantly on the high-affinity GLUT1 transporter (Km = 1–2 mM (60, 61); and the rate-limiting enzyme glucokinase (GCK) to secrete insulin through the canonical consensus model of mitochondria and glycolysis cooperation in GSIS (62). Such a shift allows mature β-cells to efficiently sense and respond to glucose levels for insulin secretion. Moreover, GLUT1 availability at the plasma membrane of β-cells in response to glucose that triggers GSIS is governed by its expression (63). Thus, the downregulated expression of HK1 and HK2 at stage 6 was concomitant with the upregulation of GLUT1 and GCK mRNA associated with a glucose-responsive phenotype (i.e. functionality).

It is important to highlight that our protocol generated 70% of PP cells at stage 3. Therefore, the lack of cell sorting for pure PP to appreciate the role of mitochondrial function in the metabolic shift observed at stage 3 during the β-cell differentiation constituted one of the main limitations of this study. Another limitation of the present study is the lack of genetic or pharmacological approaches targeting specific key mitochondrial regulators (e.g., PPARγ, PGC1α, DRP1, MFNs). Thus, while our findings suggest an association between mitochondrial remodeling and the metabolic shift observed at stage 3, the direct causal contribution of these pathways remains to be determined and warrants further study. Also, Also, quantification of mtDNA and a Seahorse assay to simultaneously measure mitochondrial respiration and glycolysis—by analyzing the oxygen consumption rate (OCR) and extracellular acidification rate (ECAR) would provide a more comprehensive view of mitochondrial health and activity.

## 5 Conclusion

Mitochondrial fitness (dynamics, energy production and signaling) plays a key regulatory role in stem cell differentiation and lineage commitment. Following a direct differentiation protocol using both HUES8 and iPSC824 stem cell lines into β-cell organoids, our results demonstrate for the first time that the metabolic switch from glycolysis to OXPHOS occurs at pancreatic progenitor stage (e.g. stage 3) as evidenced by increased mitochondrial intensity and activity at this stage. This is associated with (i-) enhanced mitochondrial biogenesis and dynamics (ii-) mitochondrial structural and organizational remodeling (from round shaped to elongated and well-defined cristae) (iii-) upregulation of genes and proteins involved in mitochondrial function (iv) increased contribution of OXPHOS to total ATP production (v-) downregulation of “disallowed” glycolytic genes and proteins. Overall, these findings indicate that mitochondrial biogenesis, remodeling, and function play an important role in the metabolic shift that occurs at the pancreatic progenitor stage of the differentiation, thereby promoting the generation of glucose-responsive β-cell organoids.

## Data Availability

The raw data supporting the conclusions of this article will be made available by the authors, without undue reservation.
