# Supplementary material for: Mitochondrial development and remodeling occur at pancreatic progenitor stage during induction of stem cell-derived islet organoids
Source: Front Endocrinol (Lausanne). 2026 Apr 23;17:1795738. doi: 10.3389/fendo.2026.1795738 (PMC13151152; doi:10.3389/fendo.2026.1795738)
Supplement: Supplementary file 1 [file Presentation1.pdf]

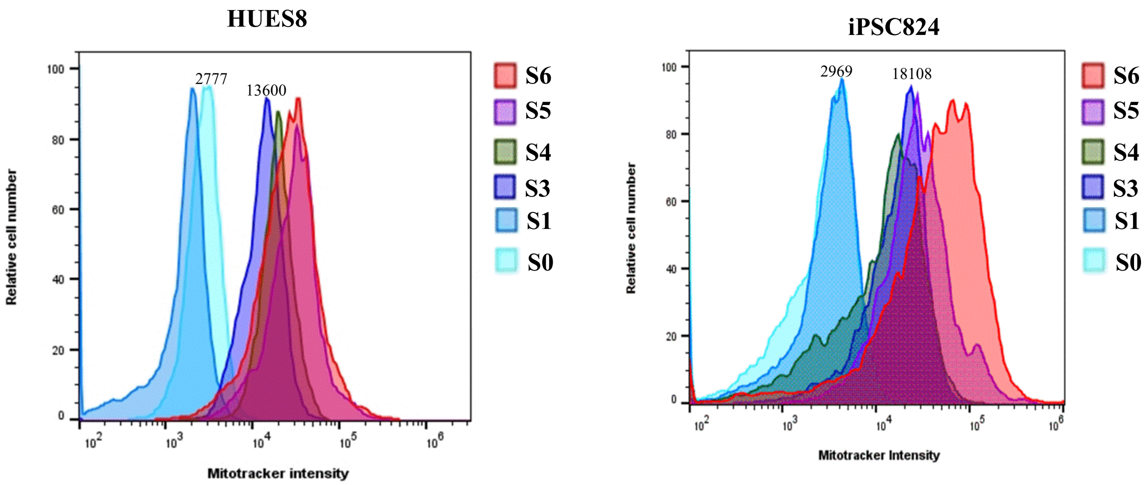

Supplementary figure 1: Mitochondrial intensity across different stages (S0, S3 and S6) of β-cell differentiation.

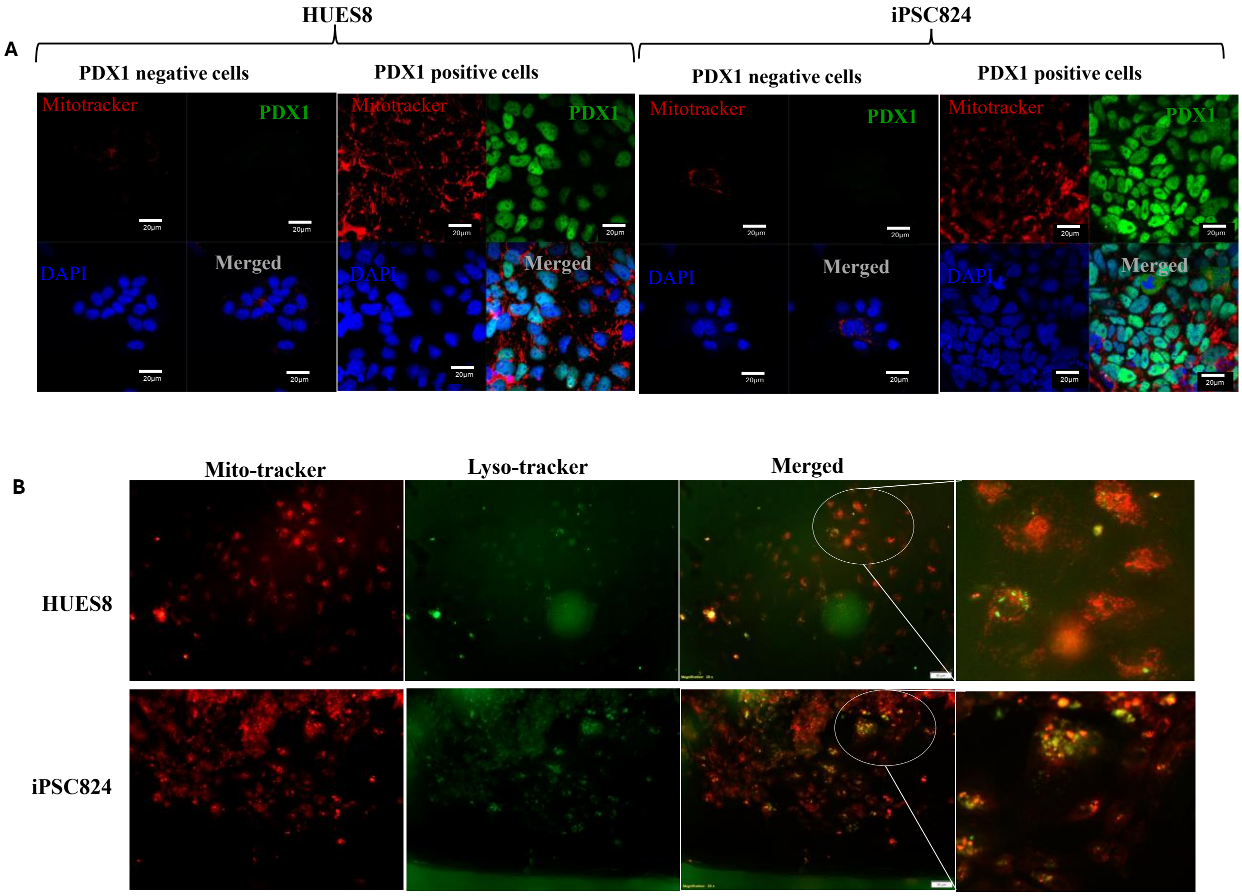

Supplementary figure 2: Representative immunofluorescence images showing co-staining of PDX1 and active mitochondrial structure using MitoTracker Deep Red (Cat no:M22426) and co-localization of mitochondria with lysosomes at stage 3 in both HUES8 and iPSC824.

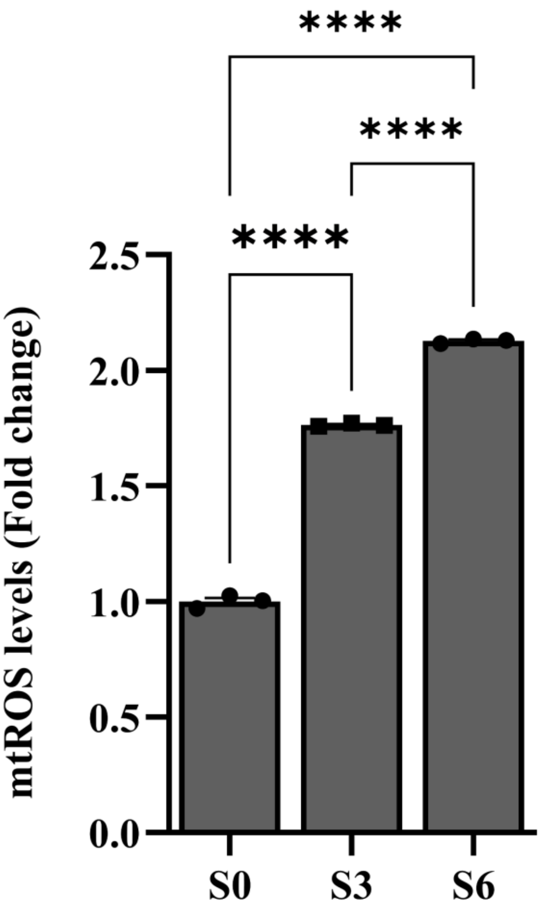

Supplementary figure 3: **Mitochondrial ROS levels across different stages (S0, S3 and S6) of β-cell differentiation.** Representative flow cytometry plots of mitochondrial ROS levels in HUES8 at different stages (S0, S3 and S6) of β-cell differentiation. The levels of mtROS were evaluated using MitoSOX™ Red mitochondrial superoxide indicators (M36007). The median flow intensity value was used as a proxy measure of mtROS. Data are representative of 3 independent experiments.
